# Hospital Equipment in War-Time

**Published:** 1916-02-05

**Authors:** 


### THE EDITOR'S LETTER-BOX.

### Hospital Equipment in War-Time.

*To the Editor of* The Hospital.

Sir,—I notice that the secretary of a London children's hospital appeals to the public to replenish the linen cupboards by gifts in kind, declaring that the new stock, that would cost normally about £250, would cost now £500, and that "to make these purchases now would almost seem like wasting the subscribers' money."

The worst of Mr. T. Glenton-Kerr's estimate is that it approximates to the truth, and promises to become as true literally as it is true substantially. Much depends on what one buys and when one bought last. Medium-quality blankets that have been made and sold at less than 1s. 3d. per lb. are at 2s. 10d. to-day. Union blankets, procurable at one time at 11½d., are now 2s. 5d., and it is none too certain that prices are at the top. Sheets, calicoes, and cottons in general have advanced in price rapidly in the last three months, and in the present sellers are avoiding commitments. As the raw cotton that was 4½d. per lb. twelve months since is 7½d. to 8d. to-day, the main factor in the rise is not obscure. The true linens, and in especial heavy goods made from flax, are very nearly twice their pre-war prices.

The causes of these disconcerting movements are perhaps less interesting than their effects, but they can be briefly stated: A large increase in the consumption of wool for the purposes of the campaign; a rising demand for cotton, with extraordinary increases in the cost of its shipment; the occupation by the enemy of the Belgian flax-growing country, and the impediments in the way of the shipment of Russian flax. The doubling of the cost of fuel and of most manufacturing stores; the depletion of labour and the advance in wages of all kinds add their influences. And doubtless most trading people look for better profits in war than in peace.

Nobody can answer how it is all to end, or predict with any certainty how soon blankets and sheets and towels will return to their old values. Mr. Glenton-Kerr's assumption appears to be that the advance is strictly temporary—a thing that came with the war and will pass away with it. The idea is an attractive one, but dangerous, and those who have to deal with purchasing will not be at all imprudent in reckoning on a continuance of high prices for a solid year longer than the duration of the war. There is no security that the market will improve by waiting, and, apart from a perilous belief in the impermanence of values, there can hardly be any question of "wasting subscribers' money" in buying the best articles to the best advantage. One of the hard facts for everyone to digest is that money-values have changed, and are under no obligation to change back again suddenly. J. A. H.

